# Enhanced decomposition of laminated ammonium perchlorate composite

**DOI:** 10.1038/s41598-021-01994-w

**Published:** 2021-11-19

**Authors:** Shuji Wang, Xueyong Guo, Wanjun Zhao, Hua Fang, Chengcheng Wu, Di Wang

**Affiliations:** State Key Laboratory of Explosion Science and Technology, Beijing Institute of Technology, Beijing, China

**Keywords:** Chemistry, Materials for energy and catalysis

## Abstract

In order to improve the thermal decomposition performances of ammonium perchlorate (AP), the laminated AP composite was prepared by ice-template induced self-assembly method. In this study, Iron-Konjac glucomannan (Fe^3+^-KGM) hydrosol rich in AP was selected as the freezing precursor. Through directional freezing of precursor and recrystallization of AP molecules, the laminated AP composite was obtained. The results showed that the thickness of the lamellar composite structure is about 10 to 30 μm, and the recrystallized AP particles are uniformly dispersed in the gel system. The oxygen bomb test results show that the micro-/nano-layered structure can significantly improve the sample’s combustion heat value. Thermal analyses indicated that with the increasing Fe^3+^ content, the peak exothermic temperature of lamellar AP composite at different heating rates both showed a decreasing trend. With 10 wt% Fe(NO_3_)_3_·9H_2_O added, the decomposition peak temperature decreased from 433.0 to 336.2 °C at a heating rate of 5 °C/min, and the apparent activation energy (*E*_*a*_) decreased dramatically from 334.1 kJ/mol to 255.4 kJ/mol. A possible catalytic thermal decomposition mechanism of lamellar AP composite catalyzed by Fe^3+^ was proposed. This work is beneficial to the structural design of other energetic materials.

## Introduction

Ammonium perchlorate (NH_4_ClO_4_, AP) has been widely used in propellants and polymer-bonded explosive (PBX) due to its high oxygen content, high density, large amounts of gas generation, simple preparation process and no heavy metal ions^1,2^. The proportion of AP in solid propellants is so high that in some formulations, some of which can even reach more than 70%^3^. Therefore, the performance of AP directly affects the performance of solid propellant and PBX^4–6^.

Thermal decomposition is the premise of combustion and detonation of AP^7^. Therefore, the research on the thermal decomposition performance of AP is the basis of improving the combustion behavior of AP-containing composite propellant and the detonation performance of PBX^8–10^. Tremendous researches have been conducted for improving the decomposition of AP, and the researches can be mainly divided into two categories: one is to design the structure of AP to improve its decomposition performance^11–14^, and the other is to catalyze the decomposition process of AP via incorporating catalyst^15–17^. The former mainly includes the porous, ultra-fine and spherical f AP. Since when the specific surface area of AP increases, more gas and heat are released per unit time, which makes the first stage of AP decomposition complete earlier, thus resulting in the better decomposition performance of AP^18^.

Catalysts can obviously change the decomposition performance of ammonium perchlorate. At present, the most studied catalysts are inorganic nonmetal (such as carbon nitride^19^, carbon based materials^20^, etc.), inorganic metal elements (such as aluminum powder^21^, copper powder, nickel copper alloy powder^22^, etc.) and metal oxides^23–32^. The principle of catalysis includes electron transfer theory^33^, proton transfer theory^7^, and energy band theory^34^. However, the agglomeration and uneven dispersion of the catalysts affect their catalytic effect significantly^35^. Therefore, promoting the dispersion uniformity of catalysts to increase the contact area with ammonium perchlorate could play a key role in enhancing the decomposition performance of ammonium perchlorate^36–40^. The fabrication of new structural materials by ice template method has been widely concerned by researchers in recent ten years, and more and more achievements have been reported^41–45^. However, the application of ice template method in the structural design of energetic materials is rarely studied.

Inspired by this, AP composites with micro-/nano-scale layered structure were prepared by ice template method. Its structure and thermal decomposition properties were characterized. The combustion heat value were tested and the kinetic parameters were calculated. On this basis, a possible catalytic mechanism of thermal decomposition of layered AP composite was proposed. This work provides a new idea for the structural design of composite energetic materials.

## Experimental

### Materials and method

**Figure 1:**
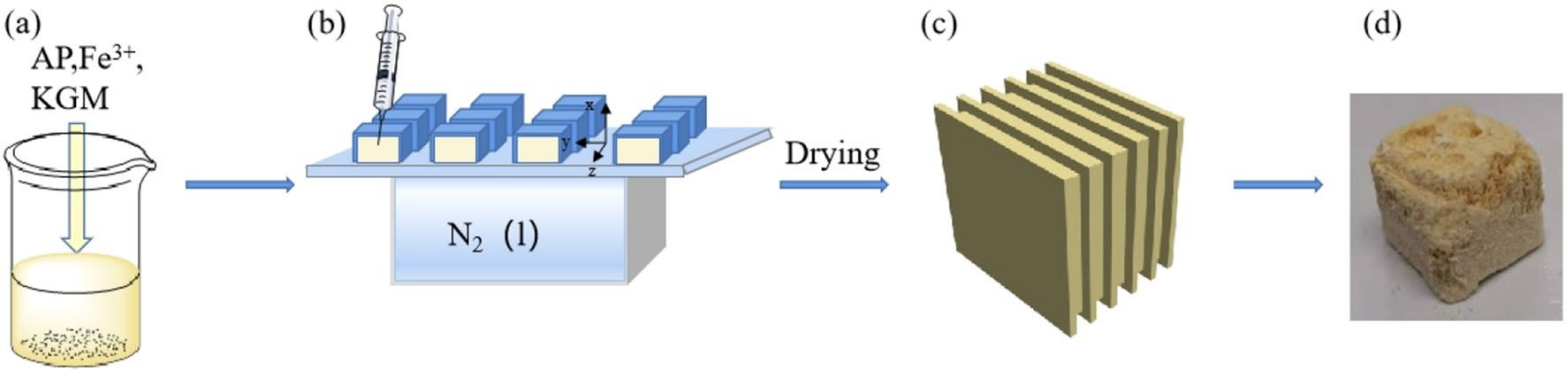
(**a-d**) Schematic illustration of preparation process of laminated AP composite.

Raw AP was provided by Liaoning Qingyang Chemical Industry Co., Ltd. Fe (NO_3_)_3_·9H_2_O and Konjac glucomannan were purchased from Shanghai Aladdin Biochemical Technology Co., Ltd. The laminated AP composite was prepared by ice-template induced self-assembly method. The process of self-assembly is shown in Fig. 1. Firstly, 2 g AP with Fe(NO_3_)_3_·9H_2_O at different ratios were added into 20 ml deionized water. Then, AP was dissolved by heating at 40 °C and stirring at 200 rpm. Then 0.2 g konjac glucomannan was added into the solution, followed by stirring 30 min to obtain sol. The template was precooled with liquid nitrogen, and then the AP sol containing catalyst was transferred to the mold on the template. Liquid nitrogen was continuously added to solidify the sol in the mold, and then the mold was quickly transferred to the tray in the drying chamber of the freeze dryer. The laminated AP composite can be obtained by vacuum freeze-drying for 48 h.

### Characterization and testing

Field-emission scanning electron microscope (FESEM, Hitachi, Japan) was used to characterize the morphology, element mapping (EDS) and contents of the laminated AP composite. X-ray diffractometer (XRD, Bruker, Germany) was used to collect the XRD pattern of as-obtained samples. X-ray photoelectron spectra (XPS) were collected by an ESCALAB 250Xi photoelectron spectrometer (Thermo Fisher Scientific, USA). An BCA500 automatic oxygen bomb calorimeter was used to test the combustion heat value (IDEA SCIENCE, USA). The thermal decomposition properties of the samples were characterized by differential scanning calorimetry (TG-DSC, Netzsch, Germany) with the different heating rates (5, 10, 15, and 20 °C/min) in a Ar atmosphere over the temperature ranged from 25–600 °C.

### Results and discussion

**Figure 2:**
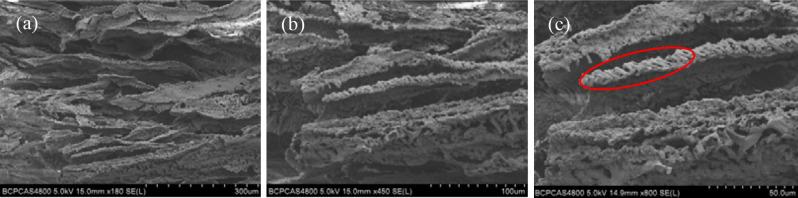
SEM images of cross sections of laminated AP composite at different magnifications.

Figure 2 shows the cross-section morphology of the laminated AP composite, which demonstrates the regularly distributed structure and indicates that the ice-template induced self-assembly method can be used to prepare the laminated-structure composite. It can be seen from Fig. 2b that the delamination between the composite layers are obviously, the gap size was between 100–300 μm, and the thickness of the single layer is 50–200 μm. By further magnification, it can be seen from Fig. 2c that AP crystals are evenly distributed in the composite structure.

**Figure 3:**
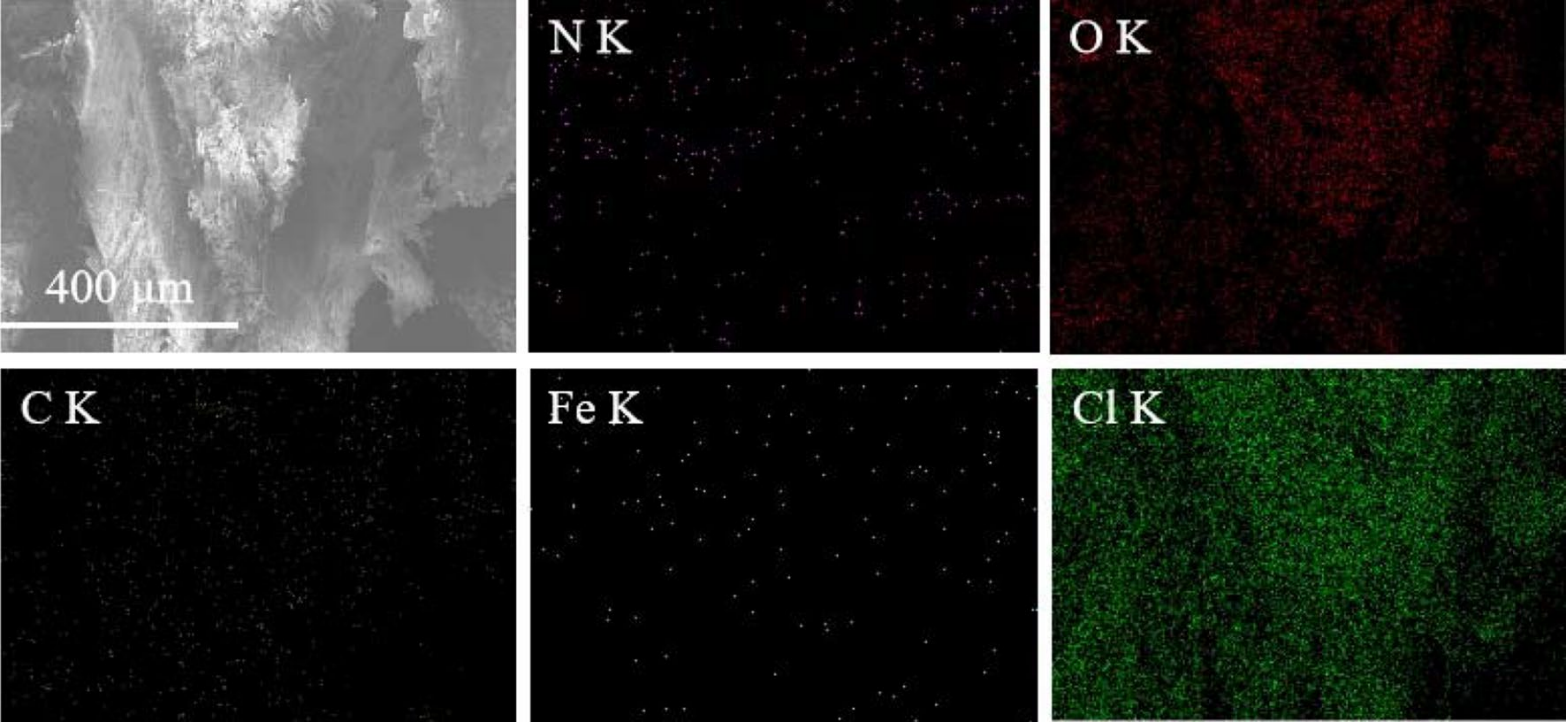
SEM images of laminated AP composite and corresponding EDS results.

**Figure 4:**
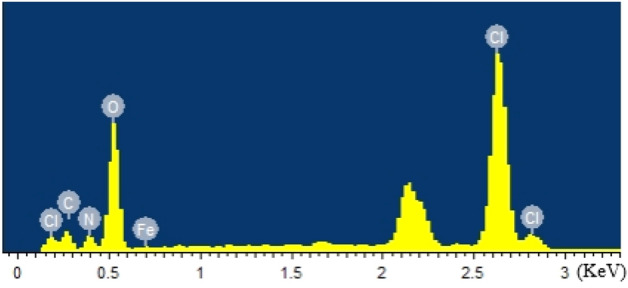
Element distributions of laminated AP composite from EDS results.

The element distribution of the sample was studied by EDS and the distribution of elements such as Cl, N, O, C and Fe are shown in Fig. 3 and Fig. 4. The element mapping of Cl and N shows that AP distributes evenly in the composite. Table 1 shows the element information where the element contents of C, N, O, Cl and Fe are 15.43, 13.70, 45.80, 23.74 and 1.33 wt%, respectively. From this, the ratio of AP to Fe content can be inferred. It can be seen from Fig. 3 that all elements are evenly distributed in the composite system, which reveals that the Fe-based catalyst can be well distributed on the surface and interface of AP, thus increasing the contact area between AP and Fe^3+^-KGM.

**Table 1:** EDS element contents of laminated AP composite.

Element	Weight (wt.%)	Atom (at.%)
C K	15.43	22.07
N K	13.70	16.81
O K	45.80	49.19
Cl K	23.74	11.51
Fe K	1.33	0.41
Total	100.00	100

**Figure 5:**
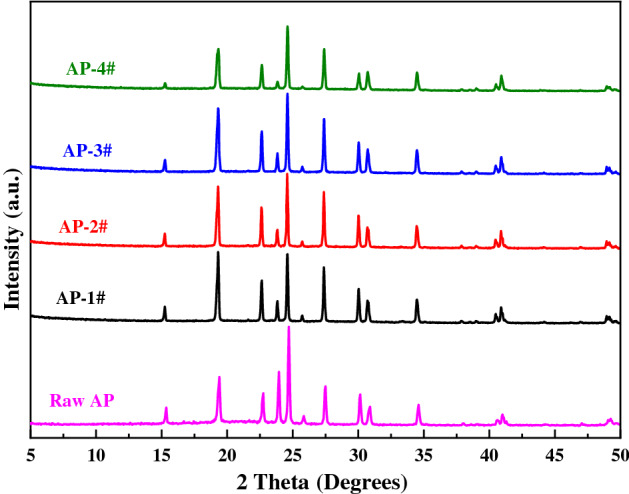
XRD patterns of raw AP and laminated AP composite samples.

The structure of raw AP and laminated AP composite (AP-1# without Fe^3+^, AP-2# with 0.125 mmol Fe^3+^, AP-3# with 0.25 mmol Fe^3+^, AP-4# with 0.5 mmol Fe^3+^) prepared by ice-template induced self-assembly method were further analyzed by XRD. The X-ray diffraction patterns are shown in Fig. 5. It can be seen from Fig. 5 that there is no obvious difference between the diffraction pattern of raw AP and the laminated AP composite. The diffraction peaks of each sample at 15.4°, 19.4°, 22.7°, 23.9°, 24.7°, 27.4°, 30.0° and 34.6° are correspond to crystal planes (101), (011), (201), ( 002), (210), (211), (112) and (401) of orthorhombic AP crystal, respectively. Since Fe^3+^-KGM sol exists in an amorphous form in the composite system, no relevant diffraction peaks are detected in the laminated AP composite. The results show that the laminated AP composite obtained by ice-template induced self-assembly method does not change the original phase composition of AP, so it has no effect on its composition and thermodynamic behavior.

**Figure 6:**
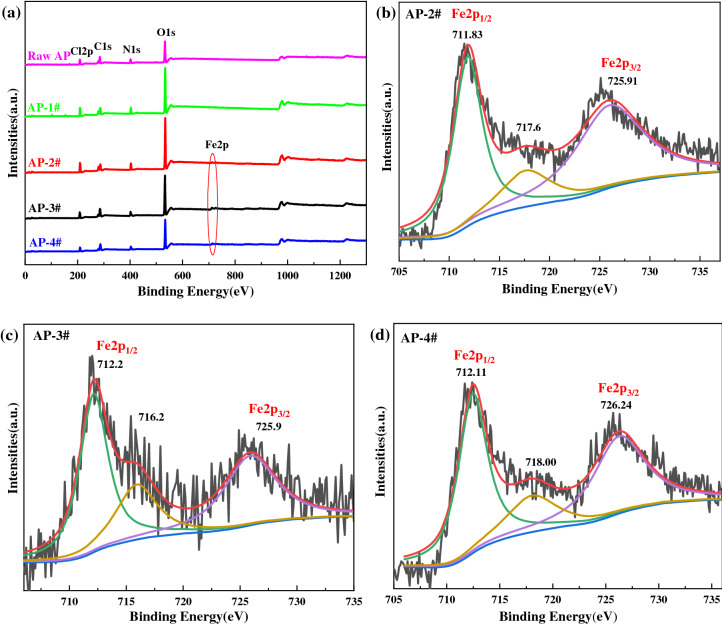
X-ray photoelectron spectra (XPS) and Fe2p XPS of laminated AP samples.

In addition, X-ray photoelectron spectroscopy (XPS) was used to characterize the distribution and state of elements in the samples. The XPS spectra of the samples are shown in Fig. 6. It can be seen from Fig. 6a that all samples contain C, N, O and Cl, and AP-2#—4# samples contain a small amount of Fe. The XPS spectrum of Fe2p can be divided into three peaks. The peak between 711–713 eV correspond to Fe2p1/2 and the peak between 725–726 eV correspond to Fe2p3/2. The satellite peak obtained at 716–718 eV are clearly distinguishable and do not overlap with Fe2p3/2 or Fe2p1/2 peaks, which is consistent with the results reported in the literature^46^.

**Figure 7:**
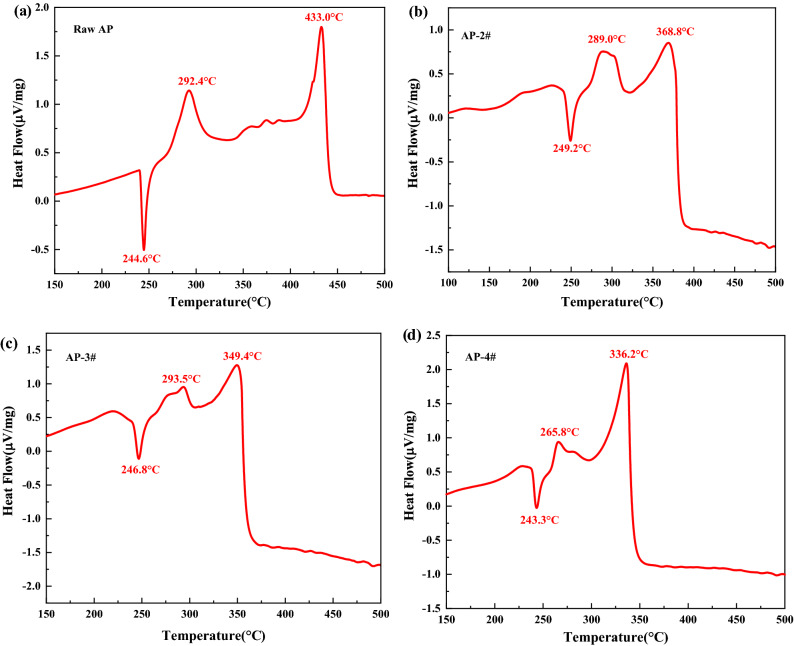
DSC curves of raw AP and laminated AP composite samples.

Furthermore, the thermal decomposition performances of raw AP and laminated AP composite were studied by differential scanning calorimetry (DSC). The thermal decomposition curves of each sample are shown in Fig. 7. It can be seen from Fig. 7a that there are three peak temperatures in the DSC curves of raw AP at 244.6 °C, 292.4 °C and 433.0 °C. The endothermic peak at 244.6 °C indicates the phase transition of AP from orthorhombic to cubic. The exothermic peak temperatures at 292.4 °C and 433.0 °C indicate that the decomposition of AP can be divided into two stages: low temperature decomposition stage and high temperature decomposition stage. Figure 7b-7d demonstrates that the DSC curves of laminated AP composite prepared by ice-template induced self-assembly strategy also has three peak temperatures. The endothermic peaks of laminated AP composite at 249.2 °C, 246.8 °C and 243.3 °C were observed, which corresponded to the crystal transformation of raw AP. At the same time, it can be found that the addition of Fe^3+^ makes the thermal decomposition peak temperature of laminated AP composite decrease, as the Fe^3+^ content in the laminated AP composite increases, the decomposition peak temperature is significantly lower than that of the raw AP. When the content of Fe^3+^ is 0.5 mmol, the decomposition peak temperature of laminated AP composite is 265.8 °C in the first stage and 336.2 °C in the second stage, which is 26.6 °C and 96.8 °C lower than that of raw AP, respectively. The lower the peak temperature of high temperature decomposition, the more concentrated the decomposition heat of AP, which result in higher heat release of the composite.

**Figure 8:**
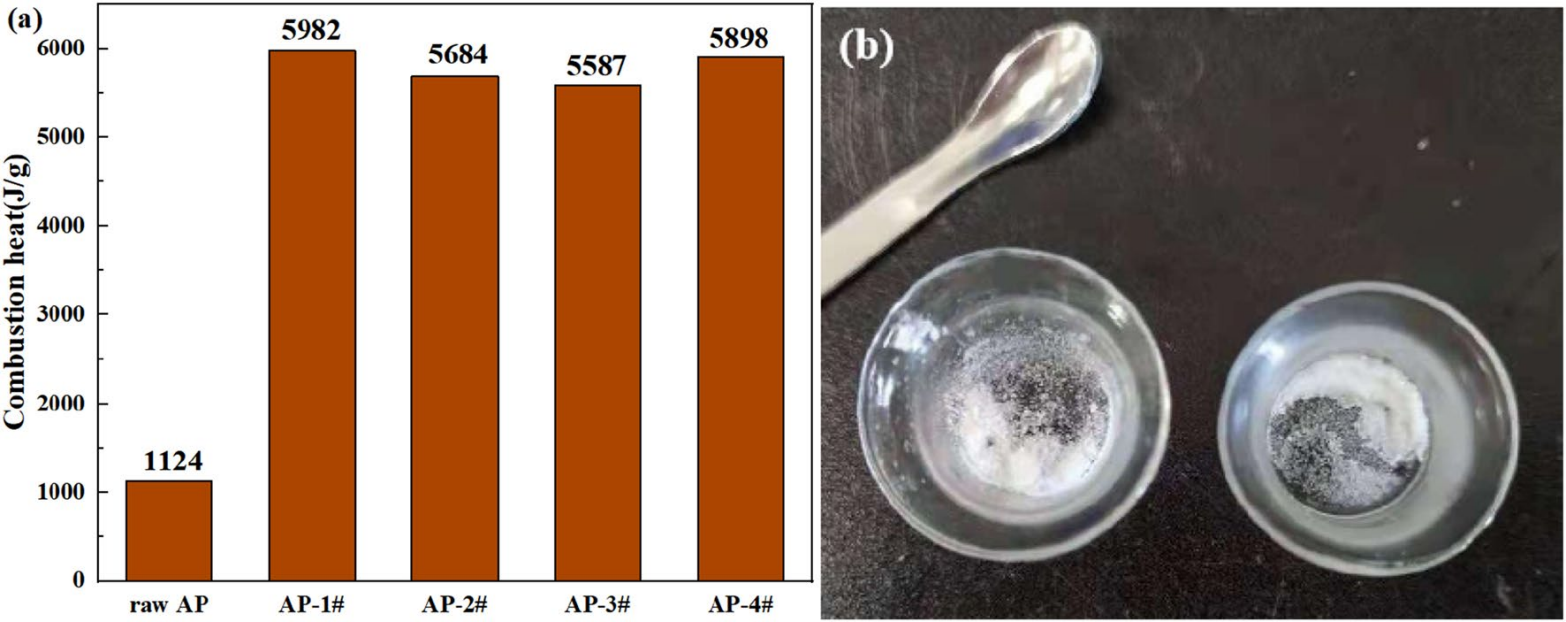
Combustion heat of the sample and incomplete combustion of raw AP.

An automatic oxygen bomb calorimeter was used to test the combustion heat of the sample. The sample mass in each test is 0.2 ± 0.0002 g, and the oxygen pressure in the oxygen bomb is 3.0 ± 0.1 MPa. Each sample was tested 3 times in parallel and the average value was taken. The results are shown in Fig. 8.

It can be seen from Fig. 8a that the combustion heat value of layered AP sample is as high as 5587–5982 J/g, which is about 5–6 times that of raw AP. As can be seen from Fig. 8b, the raw AP sample is not completely burned in the oxygen bomb because of its low calorific value, so the combustion heat value is low. The comparison of the results shows that the micro-nano layered structure can effectively improve the combustion performance of AP and greatly increase the combustion heat value due to its larger specific surface area.

**Figure 9:**
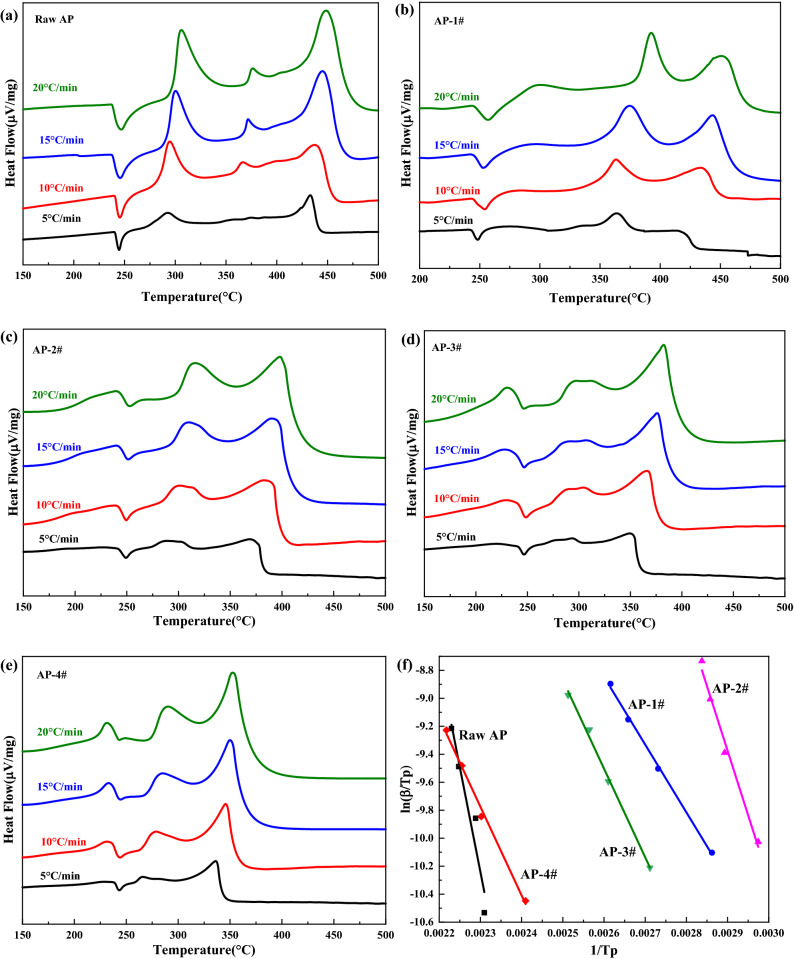
DSC curves of raw AP and laminated AP composite at different heating rates, and the dependence of ln (β/T_p_^2^) on 1/T_p_ for above samples.

The thermal decomposition parameters of raw AP and laminated AP composite were calculated by DSC curves at different heating rates (5, 10, 15, and 20 °C/min). The DSC curves at different heating rates are shown in Fig. 9. According to the relationship between exothermic peak temperature and heating rate, the thermodynamic parameters of the decomposition of raw AP and laminated AP composite (AP-1#, AP-2#, AP-3#, AP-4#) with different Fe^3+^ contents can be calculated, and the activation energy of the reaction was calculated by Kissinger equation (Eq. 1).

1 $$\mathrm{ln}\frac{\beta }{{T}_{p}^{2}}=\mathrm{ln}\frac{AR}{Ea}-\frac{Ea}{R{T}_{p}}$$

where β represents the heating rate ( °C/min), Tp represents the exothermic peak temperature. A, R and Ea represent the pre-exponential factor, gas constant and apparent activation energy (J/mol), respectively.

The kinetic parameters of raw AP and laminated AP composite were calculated based on the DSC curves, and the results are shown in Table 2. It can be seen from Table 2 that the activation energy of raw AP is 334.1 kJ/mol, which indicates that it has good thermal stability. The activation energy of laminated AP composite prepared by ice-template induced self-assembly method is obviously lower than raw AP. The activation energy of AP-4# thermal decomposition is reduced to 129.3 kJ/mol in the presence of 0.5 mmol Fe^3+^. It shows that the unique laminated structure can reduce the thermal decomposition temperature and the activation energy of AP.

**Table 2:** The kinetic parameters for thermal decomposition of obtained samples.

Sample	*T*_p_ (°C)				Ea (kJ/mol)	lg*A* (1/min)
	5	10	15	20		
Raw AP	433.0	437.0	445.0	448.5	334.1	22.6091
AP-1#	415.0	435.4	442.5	450.0	152.1	9.042
AP-2#	368.8	384.1	389.9	401.0	148.5	9.659
AP-3#	349.4	366.1	376.1	382.3	131.1	8.526
AP-4#	336.2	345.7	352.4	367.8	129.3	8.702

**Figure 10:**
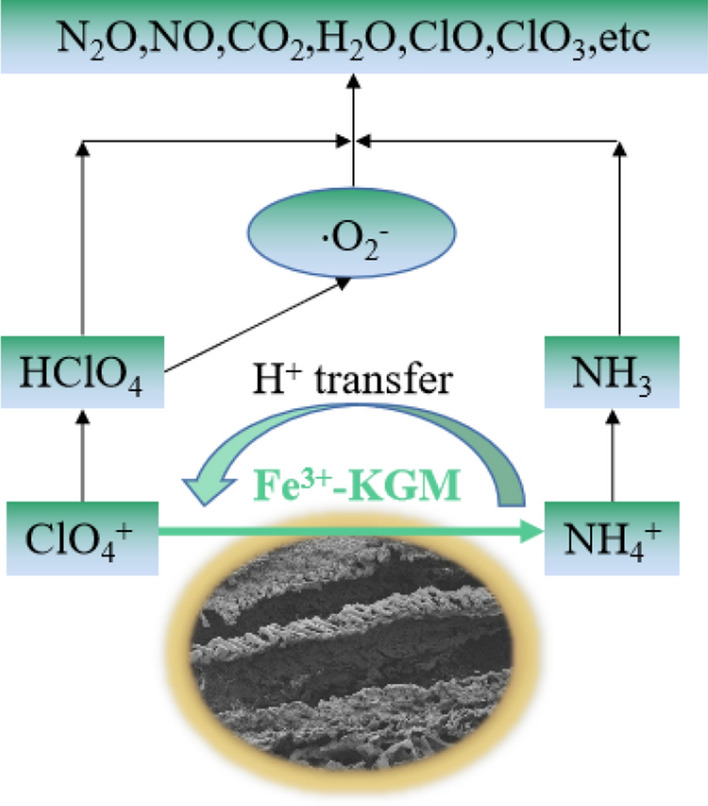
Schematic of the thermal decomposition process of laminated AP composite.

Based on the above experimental results, a possible catalytic mechanism for the thermal decomposition of laminated AP was proposed and shown in Fig. 10. With the increase of temperature, the KGM polymer gel skeleton decomposed firstly. Fe^3+^ also participates in the decomposition process of KGM molecules, and Fe-based oxides are formed on the surface and inside of AP particles. With continuous heating, nano-Fe-based oxides exhibit excellent heat and mass transfer performance on the surface and inside of AP particles, which promotes proton transfer from H of NH_4_^+^ to O of ClO_4_^-^. Superoxide anion radicals are more easily generated and continue to react with the system to form gaseous products, such as CO_2_, NO_2_, H_2_O and ClO. A large number of distributed nano-Fe-based oxides have a high catalytic contact area,which can promote the thermal decomposition of AP and make the laminated AP composite decompose rapidly at low temperature. The carbonized KGM polymer residue (the main ingredient is carbon) was further oxidized in the decomposition reaction process, which increased the heat release of the composites.

## Conclusions

In summary, the laminated AP composite with good thermal decomposition performance was prepared by ice-template induced self-assembly method. The morphological characterization results show that the single layer thickness of the laminated AP composite is between 50 and 200 μm, and the AP particles are uniformly distributed. Thermal decomposition performance tests show that the thermal decomposition peak temperature and activation energy of laminated AP composite are significantly lower than that of raw AP. When the Fe^3+^ content is 0.5 mmol, the activation energy of AP-4# is 129.3 kJ/mol, which is 204.8 kJ/mol lower than that of raw AP (334.1 kJ/mol). The possible catalytic mechanism of the laminated AP composite thermal decomposition was proposed. This work provides a new idea for the structural design of energetic materials.
